# Indoor Localization within Multi-Story Buildings Using MAC and RSSI Fingerprint Vectors

**DOI:** 10.3390/s19112433

**Published:** 2019-05-28

**Authors:** Litao Han, Li Jiang, Qiaoli Kong, Ji Wang, Aiguo Zhang, Shiming Song

**Affiliations:** 1 College of Geomatics, Shandong University of Science and Technology, Qingdao 266590, China; qiaolikong@sdust.edu.cn (Q.K.); wj10946@163.com (J.W.); s940087422@163.com (S.S.); 2 College of Computer and Information Engineering, Xiamen Institute of Technology, Xiamen 361024, China; zhangaiguo@xmut.edu.cn

**Keywords:** indoor localization, media access control address, floor identification, multi-story buildings, fingerprint localization

## Abstract

For existing wireless network devices and smart phones to achieve available positioning accuracy easily, fingerprint localization is widely used in indoor positioning, which depends on the differences of the Received Signal Strength Indicator (RSSI) from the Wireless Local Area Network (WLAN) in different places. Currently, most researchers pay more attention to the improvement of online positioning algorithms using RSSI values, while few focus on the MAC (media access control) addresses received from the WLAN. Accordingly, we attempt to integrate MAC addresses and RSSI values simultaneously in order to realize indoor localization within multi-story buildings. A novel approach to indoor positioning within multi-story buildings is presented in this article, which includes two steps: firstly, to identify the floor using the difference of received MAC addresses in different floors; secondly, to implement further localization on the same floor. Meanwhile, clustering operation using MAC addresses as the clustering index is introduced in the online positioning phase to improve the efficiency and accuracy of indoor positioning. Experimental results show that the proposed approach can achieve not only the precise location with the horizontal accuracy of 1.8 meters, but also the floor where the receiver is located within multi-story buildings.

## 1. Introduction

Nowadays, as many people spend about 80% of their life indoors [1], indoor location is becoming more and more important to our daily life. The Global Positioning System (GPS) is a typical localization technology that has been used in various fields. However, various indoor localization techniques are required because GPS signals cannot be received in indoor environments. How to realize high-precision and high-practical indoor positioning technology has become a research hotspot in academia and industry [2]. Currently, the popular indoor positioning technologies can be classified into three categories: the first is indoor positioning technologies based on mobile phone sensors [3,4] such as accelerometer, gyroscope, barometer; the second is indoor positioning technologies based on radio frequency signals [5] such as WLAN, Bluetooth, cellular mobile signals; the third is multi-source fusion localization methods [6] which integrate a variety of positioning technologies based on mobile phone platforms. Generally, indoor positioning methods based on sensors, e.g., PDR (Pedestrian Dead Reckoning) [7], are limited by the sensor accuracy of the mobile phone and the positioning principle of pedestrian calculation, so they inevitably produce accumulated errors. Although the indoor positioning method based on radio frequency signal has better positioning accuracy and low cost, its positioning accuracy is unstable [8]. Meanwhile, the multi-sensor fusion localization method, such as WiFi-PDR (Pedestrian Dead Reckoning) indoor positioning, has become a new trend [9], but the heterogeneity of all kinds of sensors makes the fusion calculation challenging. Accordingly, indoor localization dependent on WLAN is still widely used in many applications because of low cost and low difficulty [10].

Besides the plane position of moving receivers, their floor position is necessary to locate them perfectly within multi-story buildings. Some researchers have done some work on floor identification. Deng et al. [11] proposed a WLAN indoor floor identification method based on the k-means algorithm that classifies floors according to the relationship between RSSI value and distance and takes the nearest class of subordinate floors as the final result. Alex et al. [12] proposed another method based on GSM (global system for mobile communications) fingerprint to identify the floor and studied different methods to reduce the amount of fingerprint collection effectively under the condition of ensuring a high identification accuracy rate. Li et al. [13] introduced prior information, such as an indoor environment map, to help the RSSI of WLAN to realize floor identification. Literature [14] applied WKNN (Weight K-Nearest Neighbor) and neural network respectively to floor judgment, which was also based on the RSSI. In contrast to the above, [15,16] used differential barometric altimetry to assist with floor identification and achieved a judgement rate of 98%. In addition, in order to improve the matching efficiency of online positioning, different clustering algorithms were introduced [17,18,19,20,21,22,23], such as interior geometric clustering algorithm. Some researchers considered APs (access points) as Voronoi diagram’s generators to create Voronoi cells and used these cells to cluster fingerprints of database [22,23]. After that, the location area of mobile nodes was determined by using the strongest access point, and finally used the dynamic KNN (K-nearest neighbor) algorithm to implement the fingerprint clustering algorithm.

The major innovation of this article is that MAC addresses of APs are applied to indoor localization within the multi-story buildings. Firstly, MAC addresses and RSSI from WLAN in multi-floor indoor environment are collected and stored into fingerprint database. Secondly, the floor where the receivers are located is identified through matching the similarity between the received MAC address vector of receivers and that pre-stored in the fingerprint database. Thirdly, the further localization results on the same floor are obtained through the coarse positioning and the precise positioning. In the proposed method, MAC addresses are fully used and clustering reference points taking MAC as the classification index can largely reduce the search space of RSSI matching, which will improve the efficiency of RSSI matching.

The rest of the paper is organized as follows. In Section 2, the main idea and design of indoor localization of multi-story indoor environment is proposed. Section 3 shows the data collection and the establishment of fingerprint database. Section 4 gives the detailed introduction about the proposed approach. A case study used to test the proposed method is shown in Section 5. Finally, discussion and concluding remarks are given in Section 6.

## 2. Main Idea of Proposed Approach

**Figure 1:**
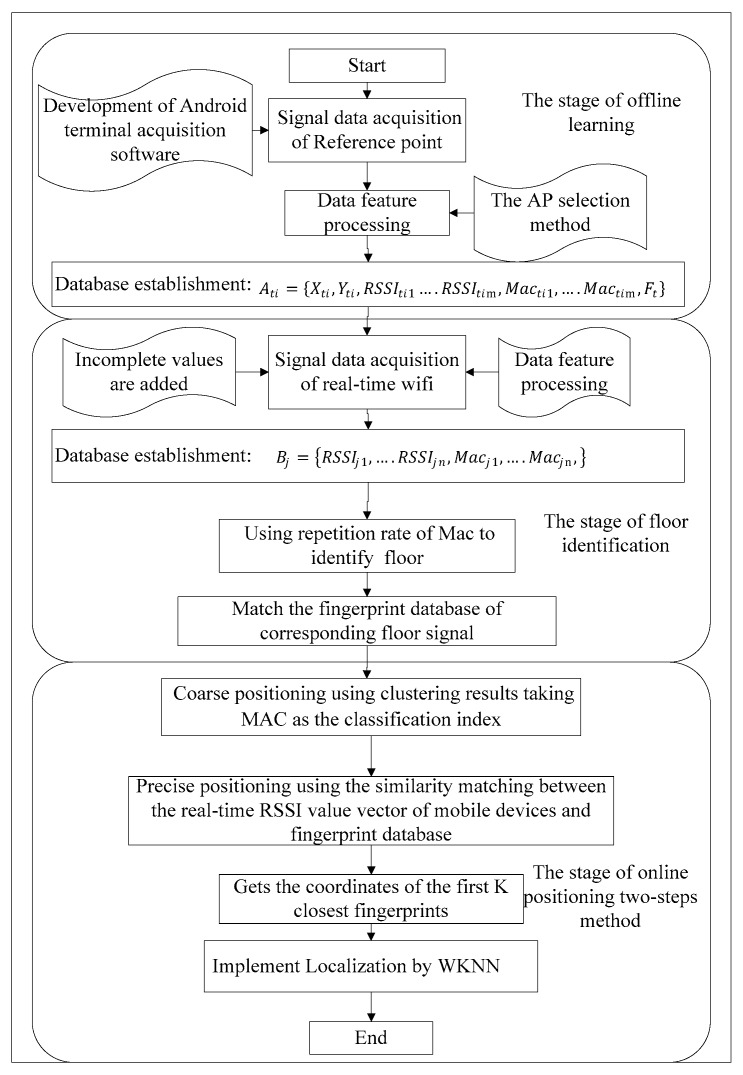
The proposed flowchart of indoor localization within multi-story buildings based on Wi-Fi.

Generally, the positioning process of wireless fingerprint localization based on WLAN is divided into two phases: the establishment of fingerprint database and real-time positioning [24]. However, considering the possibility of improving positioning precision by adding MAC addresses to the indoor localization, we proposed a novel indoor localization approach. Compared with the existing methods, the proposed one adds the floor identification phase. The detailed positioning procedure is shown in Figure 1.

The process of the proposed indoor localization approach is divided into three phases: offline phase, online floor identification phase, and online positioning phase. The third one includes clustering-based coarse positioning by MAC addresses and precise positioning by RSSI. In the offline learning phase, a number of planned reference points are laid out in the location area, and RSSI values and MAC addresses from APs are collected at those points by our developed collector on Android platform to establish the fingerprint database. After that, all reference points are classified into many sub-classes by the clustering algorithm that takes MAC addresses as the classification index. In the phase of floor identification, the repetition rate of MAC addresses is used to identify the floor where the receiver is. In the phase of two-step online positioning, firstly, coarse positioning is implemented by matching MAC addresses based on offline clustering results; secondly, precise positioning is achieved by using the similarity matching between the real-time RSSI value vector of the receivers and those pre-stored in the fingerprint database [25]. These phases above will be introduced separately in the following parts.

## 3. Data Collection and Database Establishment

### 3.1. Data Collection

It is the first step of Wi-Fi fingerprint localization to collect the signals of APs inside the buildings, which includes layout of reference points and the following collection of MACs and RSSIs of all available APs in each reference point. The interval of two neighboring reference points should be planned reasonably for it will impact the localization accuracy largely.

**Figure 2:**
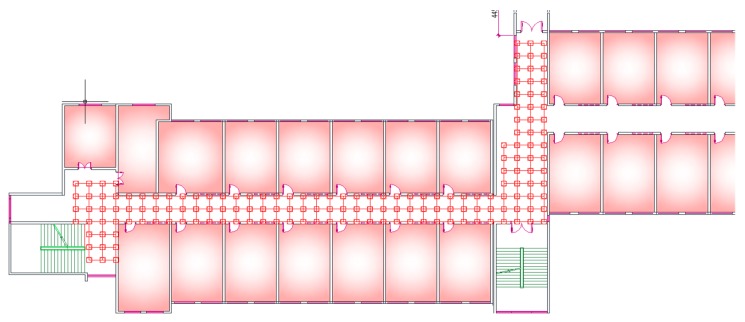
Experimental environment and the distribution of reference points on one floor.

**Figure 3:**
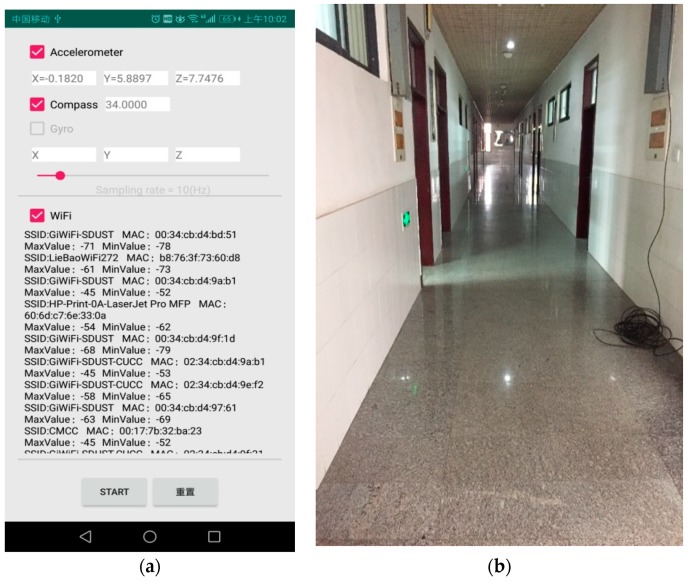
Collection of the RSSI values and MAC addresses of APs: (**a**) The interface of signal acquisition software; (**b**) The actual environment of data collection.

Our experiment was carried out in a real scenario (our office building). It directly utilized many existing Wi-Fi hotspots installed in the corridors and rooms of the building, which were specially designed for building wireless networks. The test area includes the second and third floors of the building. Figure 2 shows one of the two floors. The location testing area of each floor was divided into a number of grids. The intersection of grids was called reference points with the interval of 1.2 m, the number of which on each floor was 160 groups. Then, the information of all available APs installed in the building were collected at each reference point by a signal acquisition software that was developed on Android using the java programming language. The software was designed mainly to collect RSSIs and MACs at each reference point and to obtain the acceleration, direction and attitude of a smartphone, whose main interface was shown in Figure 3a.

When the Wi-Fi mode was checked, the information of APs could be collected that included service set identifier (SSID), MAC addresses, the lowest RSSI value and the highest RSSI value. Figure 3b shows the actual environment of data collection. RSSI of each AP changes in real time and is not a constant value. For this reason, we collected the RSSI values and MAC addresses 30 times at every reference point of two floors. The sampling frequency was 10 Hz and the sampling time was 3 s. After that, all original information was saved to support the following work.

### 3.2. Database Establishment

**Figure 4:**
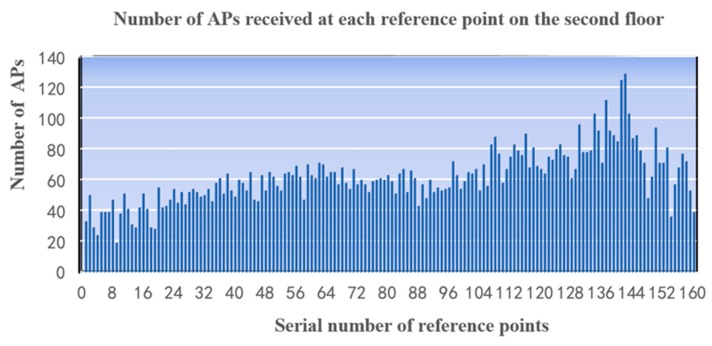
The number of APs received at each reference point on the second floor.

Due to the large quantity of APs and reference points and the times of collection, the data of MAC addresses and RSSI values are huge [26]. It’s necessary to extract eigenvalues in order to establish the fingerprinting database [27]. In order to make efficient use of the collected AP (Access Point) information, AP optimization is required [28]. The reasonable selection of APs can not only improve the accuracy of positioning, but also reduce the computation time. As dozens of APs (the statistical results are 19–129, as shown in Figure 4) can be available to the receiver at each sampling point, the top 10 APs ranking by RSSI are selected to establish the fingerprinting database. Besides having the strongest signals, the top 10 APs must have long-term and stable availability.

The specific process is as follows: for all reference points in the fingerprint database, the mean of each AP’s 30 RSSI values during the period of data collection is firstly computed, and then the fingerprints of all APs are sort in descending order based on the RSSI means, lastly the top 10 APs are selected to establish the fingerprinting database that stores RSSI values and MAC addresses of all selected APs. In order to realize the floor identification and the location on the same floor, the fingerprinting database must store horizontal position, received RSSI vector, received AC vector and floor of each reference point. Data of each reference point is stored in the database as one record. Taking data on one floor as an example, their organization is shown as Table 1.

## 4. Indoor Localization Based on MAC and RSSI 

Based on the fingerprinting database, we proposed a new approach to implement multi-floor indoor localization, which mainly includes two steps: the identification of floors and the indoor localization on the same floor. While users enter the indoor environment, the characteristic vector of signals of all accessible APs will be extracted to match the fingerprint database. Floor identification can be carried out by calculating the repetition rate between MAC addresses of received APs and those of reference points stored in the fingerprinting database. Furthermore, on the same floor, the moving receivers’ position can be obtained by matching RSSI, which includes two steps: clustering-based coarse positioning and precise positioning. Clustering-based coarse positioning divides the area where the reference points cover into many sub-regions according to the MAC repetition rate. With respect to precise positioning, the accurate position of the receivers can be achieved by WKNN positioning algorithm based on RSSI match in the sub-regions.

### 4.1. Floor Identification

**Figure 5:**
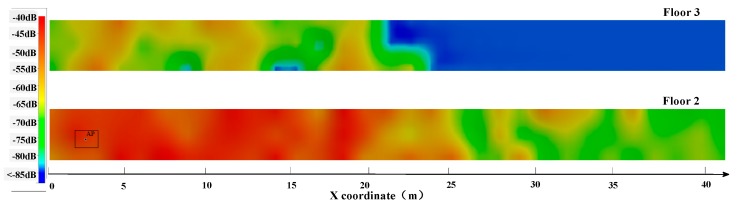
The RSSI distribution of the test AP on two different floors in which the red part indicates that the signal intensity is high.

In order to identify the floor where the receiver is, the relationship between the floor number and received signals of APs on it need to be discovered. First of all, we need to know one AP’s RSSI values distribution on different floors. Taking one AP on the west side of the second floor of the testing building as an example, marked with a black circle in Figure 5, the distribution of RSSI values on the second and third floors was tested. The vertical distance between the two floors is 3 m. The lower color strip represents the distribution of the AP’s RSSI on the second floor and the upper represents that on the third. The legend on the left represents the relationship between the color and RSSI values. According to Figure 5, we can infer that the received RSSI values of the AP are not only related to the distance away from the receiver (smartphone), but also influenced seriously by the building structure. The received signal strength (RSS) of the AP on the upper floor is obviously weaker than that on the lower floor because the AP’s signal is blocked by the building walls or floors.

**Figure 6:**
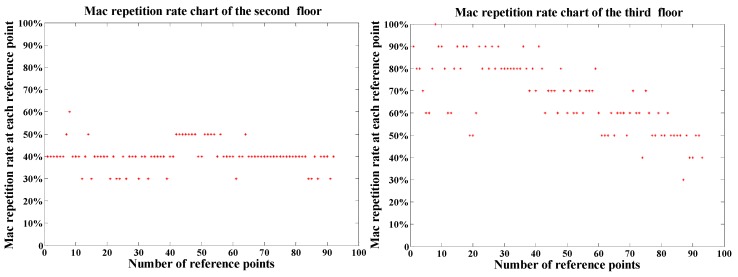
Comparison of MAC repetition rate chart of the second floor and that of the third one.

Since RSSI values have a corresponding relationship with MAC addresses and MAC addresses are more fixed than RSSI values relatively, we attempt to take the MAC address vector as the matching index instead of the RSSI value vector to identify the floor more accurately. A test point *p* was laid on the third floor, and its RSSI value and MAC addresses were received. The MAC repetition rate is calculated based on the received MAC vector of point *p* and that of each reference point. The dimension of the MAC address vector is 10. The MAC repetition rate of *p* and reference points can be obtained by:

(1) $$\begin{array}{c} C_{ti}=MACStrCmp\left(M_{pm},M_{tim}\right)/m\\ M_{tim}=\left\{Mac_{ti1},Mac_{ti2},Mac_{ti3}\cdots \cdots Mac_{tim}\right\},\;\\ M_{pm}=\left\{Mac_{p1},Mac_{p2},Mac_{p3},\cdots \cdots \cdots Mac_{pm}\right\} \end{array}$$

where t is 2 and 3, m is 10, $M_{tim}$ and $M_{pm}$ are represented as character vectors composed of MAC addresses of APs received by the receiver. MACStrCmp() is the function to get the number of MAC repetition by comparing string $M_{pm}$ with each element of $M_{tim}$. $C_{ti}$ denotes the MAC repetition rate of *p* and reference point $A_{ti}$. In order to improve the comparison efficiency of searching for the same MAC addresses in two vectors, MAC addresses expressed as strings can be represented by integers. Figure 6 shows that MAC repetition rate between the MAC address vector of *p* and that of each reference point on the second and third floors.

As shown in Figure 6, the horizontal axis denotes the number of points on the floor, and the vertical one denotes the MAC repetition rate of *p* and reference points. For *p*, the MAC repetition rate on the second floor is mostly 4, and some of them are 5, with the maximum value of 6. On the third floor where *p* is, the MAC repetition rate is mostly greater than 5, with the maximum value of 10. This experiment shows that MAC addresses received at reference points on different floors have strong correlation and obvious difference. The MAC addresses of reference points in the fingerprint database can be used to achieve the floor number where the moving receivers are. The procedure of floor identification is as follows:

1. Obtain APs’ signal collected by the receiver at real-time test point $B_{j}$, extract the characteristic values according to the method in Section 3.2 and record them in the following format:

$$\left\{RSSI_{j1},\;RSSI_{j2},\;\cdots ,\;RSSI_{jm},\;Mac_{j1},\;Mac_{j2},\cdots ,\;Mac_{jm}\right\}$$

where j denotes the numbers of real-time test points passed by the moving receiver, m is 10. $R_{ji}$ and $M_{ji}$ denote respectively the ith RSSI and the ith MAC of the top m of RSSIs and corresponding MAC addresses received at the point $B_{j}$.

2. Get the relevant information of all reference points stored in the database. The format of a piece of information is as below:

$$\left\{A_{ti},\;X_{ti},Y_{ti},\left\{RSSI_{ti1},\;RSSI_{ti2},\;\cdots ,\;RSSI_{tim}\right\},\left\{Mac_{ti1},\;Mac_{ti2},\;\cdots ,\;Mac_{tim}\right\},F_{t}\right\}$$

3. Calculate the MAC repetition rate between $B_{j}$ and all reference points to achieve floor identification. The detailed process is as follows:

(a). Firstly, the MAC repetition rates between reference points on the floor $F_{t}$ and the test point $B_{j}$ are calculated according to the following equation:

(2) $$C_{ji}\;=MACstrcmp\left(\left\{Mac_{j1},\;Mac_{j2},\cdots ,\;Mac_{jm}\right\},\left\{Mac_{ti1},\;Mac_{ti2},\;\cdots ,\;Mac_{tim}\right\}\right)/m$$

(b). Secondly, given the threshold δ of $C_{ji}$, the number of reference points where the MAC repetition rate is greater than the threshold is counted as follows:

(3) $$N\;=Num\;(C_{ji}>\delta )$$

(c). Thirdly, the proportion of reference points meeting the threshold requirement on the same floor is calculated as follows:

(4) $$P_{jt}=N/Num\left(F_{t}\right)$$

where $Num(F_{t}$) is the total number of reference points on the floor $F_{t}$.

(d). Finally, after the proportion on each floor is calculated, the floor with the maximum proportion is regarded as the floor where the point $B_{j}$ is and can be obtained by the following equation:

(5) $$Floor=index\;(max\;(P_{jt})$$

where index( ) is used to get the floor number with the maximum proportion.

### 4.2. Two-Step Positioning on The Same Floor

#### 4.2.1. Clustering-Based Coarse Positioning

**Figure 7:**
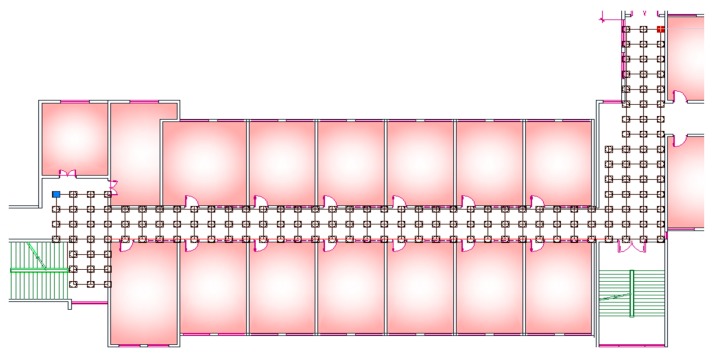
The distribution of test points and reference points: the red point is the starting point and the blue is the end.

Generally speaking, a clustering algorithm can achieve information aggregation by classifying process according to the similarity between sample points. In this paper, the clustering algorithm is used to realize the division of fingerprints and coarse location. Comparing with the clustering algorithm by RSSI, we take the MAC addresses of APs as the clustering index. In order to verify whether the Mac addresses of APs can be used as clustering index, a test was conducted in the experimental area as shown in Figure 7. The process of collecting AP’s signal starts from the westernmost point (the blue point) in the indoor space, and continues from west to east until the easternmost one (the red point). It is worth noting that reference points are numbered from the west to east.

**Figure 8:**
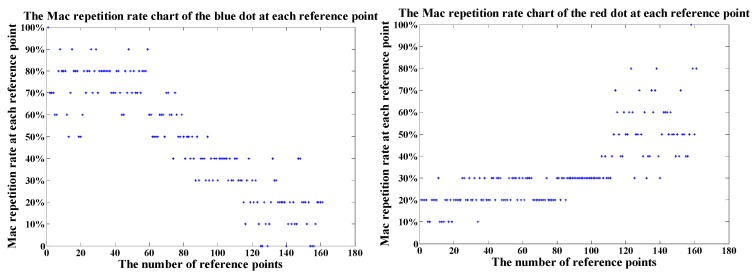
The distributions of MAC repetition rate of two test points on the same floor.

After that, the two points farthest from each other are selected as test points to calculate the MAC repetition rate between them and all reference points on the testing floor respectively. A comparison of MAC repetition rate between the two points is shown in Figure 8.

We can infer that the distribution of MAC repetition rate between the test point and the reference points has strong spatial autocorrelation. On the left chart, for example, because the blue test point is located in the west and close to the reference points numbered in the former part such as 1–100, the MAC repetition rate between the blue test point and the former part of the reference points is in upper part and it’s greater than that numbered in the later part such as 100–180. On the contrary, on the right chart, because the red test point is close to the reference points in the east and their MAC repetition rate is higher than that in the west. The distribution feature of MAC addresses can be used as clustering index to cluster the MAC Addresses of indoor APs.

As we all know, one AP’s MAC address is a fixed value as long as the receiver can get its signal. Compared with using RSSI as clustering index, using Mac address as clustering index is more stable and applicable because RSSI is easy to be influenced by the surrounding magnetic field and the ability of receiving signals of the receiver. The core work of the clustering algorithm is to determine the clustering center and the clustering number. K-Means algorithm that is used normally belongs to hard classification. However, hard classification does not take into account whether correlation between categorical data. In addition, MAC addresses between reference points have a certain correlation with distance, so that the traditional K-Means algorithm does not apply to the proposed clustering algorithm that is based on MAC repetition rate. Accordingly, we proposed an adaptive method for the clustering center selection that will choose m reference points as far as possible from each other as the clustering centers. That is, firstly, a random reference point as the first initial cluster center is chosen, the reference point farthest from the first point is selected as the center of the second initial cluster, the reference point farthest from the first two points is selected as the center of the third initial cluster, and so on, until m cluster centers are selected. The procedure of clustering reference points by MAC address is as follows:

1. Determine the clustering centers and clustering number according to the method above;

2. Calculate the MAC repetition rate between each reference point and all clustering centers as follows:

(6) $$R_{ik}=MACStrcmp\left(\left\{Mac_{k1},Mac_{k2},\ldots ,Mac_{k10}\right\},\left\{Mac_{i1},Mac_{i2},\;\ldots ,Mac_{i10}\right\}\right)/10$$

where k is the cluster number and i is the number of reference points.

3. Add the reference point i into the cluster k whose center has the highest MAC repetition rate with it. As a result, all reference points in the fingerprint database are divided into m sub- regions.

**Figure 9:**
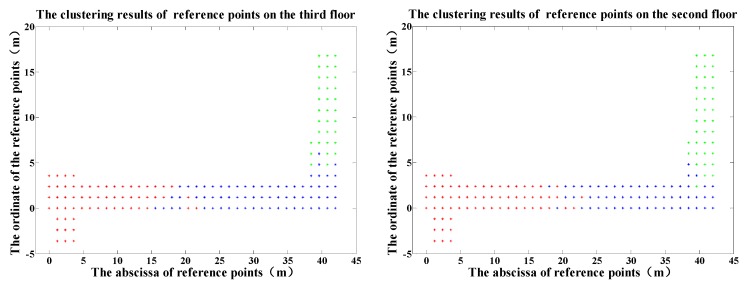
The clustered results of reference points on two floors.

According to the procedure above, clustering experiments were carried out. Figure 9 shows the clustering results of all reference points in the testing region. Reference points belonging to the same class are represented in the same color. We can see that the partition results conform basically to the spatial distribution of indoor reference points. Here, we should note that this process is done before online positioning.

After clustering reference points at the offline stage, we can use clustering results at the online stage to determine which class the test point (namely estimated point) belongs to. The procedure of clustering-based coarse positioning is as follows:

1. Calculate MAC repetition rate between the test point j and clustering centers

(7) $$Q_{\mathrm{jk}}=MACStrcmp\left(\left\{Mac_{j1},Mac_{j2},\ldots ,Mac_{j10}\right\},\left\{Mac_{k1},Mac_{k2},\ldots ,Mac_{k10}\right\}\right)/10$$

2. Find out the clustering region whose clustering center has the highest MAC repetition rate with the test point to determine which category $L_{k}$ the test point *j* belongs to.

(8) $$L_{jk}=\mathrm{index}\left(\mathrm{Max}\left(Q_{jk}\right)\right)$$

#### 4.2.2. Precise Positioning

After clustering-based coarse positioning, the test point is located in the specified sub-region. The following work is to determine its precise position in the sub-region. The WKNN algorithm based on RSSI vector is used to implement the precise positioning in the sub-region. The detailed procedure is as follows:

1. Calculate Euclidean distance between the RSSI vector of the test point j and those of reference points in the sub-region $L_{jk}$ as follows:

(9) $$D_{ji}=\sqrt{\sum_{i=1}^{n}{\left(RSSI_{j}-L_{jk}\left(RSSI_{in}\right)\right)}^{2}}$$

where j is the number of the test point and n is the number of reference points in the sub-region $L_{jk}$.

2. Select K reference points with the smallest Euclidean distance from the test point to calculate the mean of their coordinates as the final position of the receiver.

(10) (Xj,Yj)=∑i=1K1Dji∑i=1K1Dji(Xi,Yi)

## 5. Experimental Evaluation and Discussion

**Figure 10:**
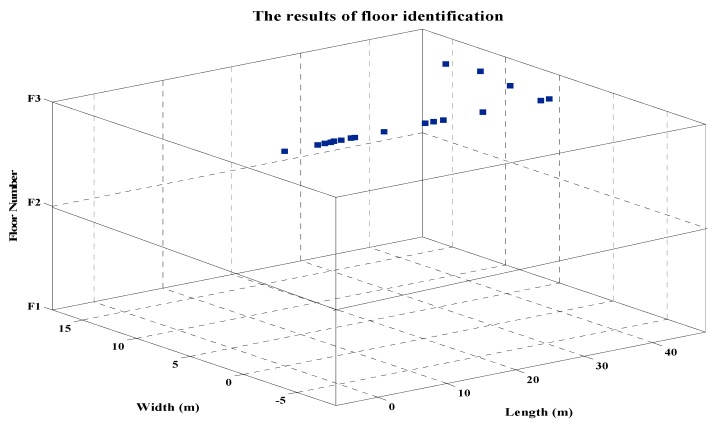
The results of floor identification.

In order to verify the proposed method of indoor three-dimensional localization, an indoor three-dimensional positioning test was carried out. We collected the RSSI and MAC information of referenced points placed from the first floor to the third floor of the teaching building J6 of College of Geomatics of Shandong University of Science and Technology. The collected data were processed according to the method introduced in Section 3, and the fingerprint database was established. And then we tested the proposed algorithm for indoor three-dimensional localization. Figure 10 shows the floor identification results of the moving receiver. As shown in Figure 10, the proposed method of floor identification based on MAC repetition rate can accurately identify the floor where the moving receiver is located (the height of each floor is 3 m).

**Figure 11:**
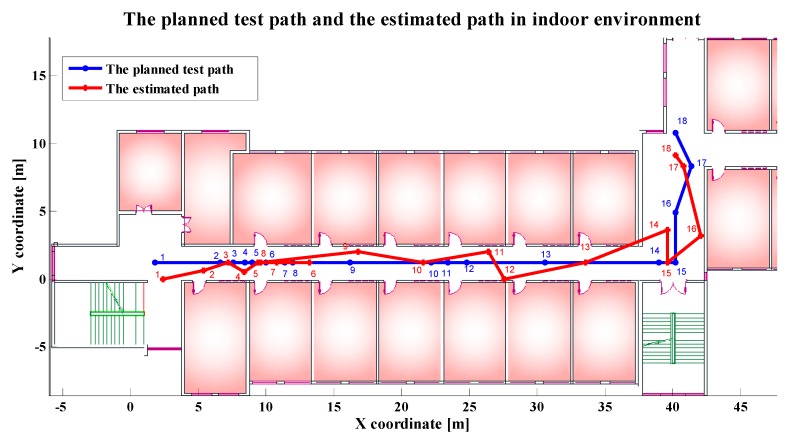
Comparison of the planned test path and the estimated path in indoor environment.

In order to get the accurate position of the moving receiver within the single floor, we used the two-step method to locate the moving receiver. Here, we sampled 18 test points along the central line of the corridor. These test points were selected in the sites where the location characteristics were more obvious, for example, the corners of floor tiles, the points aligned with the edge of the door. Half of them were densely placed at a shorter distance and half were scattered at a longer distance to make test points have a more reasonable distribution. Figure 11 shows the comparison of the planned test path and the real-time estimated path on the same floor. The blue one is the planned test path. The red is the real-time estimated path of the moving receiver. It can be seen that the orientation of the estimated path is consistent with the orientation of the test path. Table 2 shows that the average of localization errors between planned test points and real-time estimated points is 1.8 m, with a minimum of 0.4 m.

### 5.1. Threshold Determination of MAC Repetition Rate

**Figure 12:**
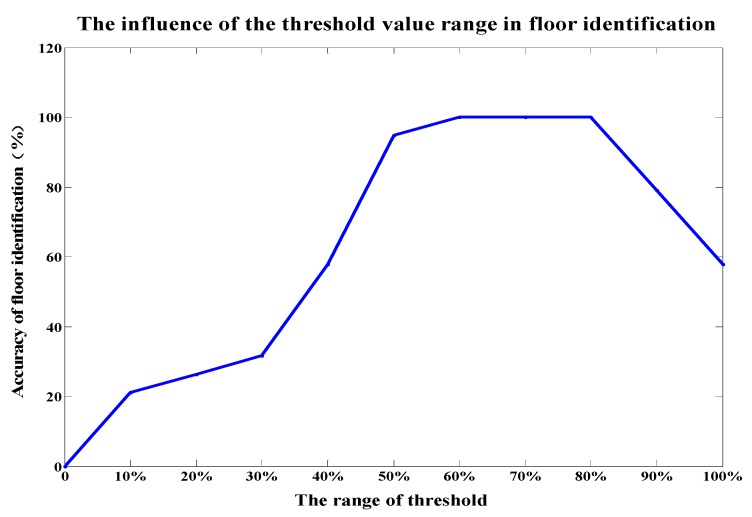
Impact of the threshold of MAC repetition rate on floor identification’s accuracy.

**Figure 13:**
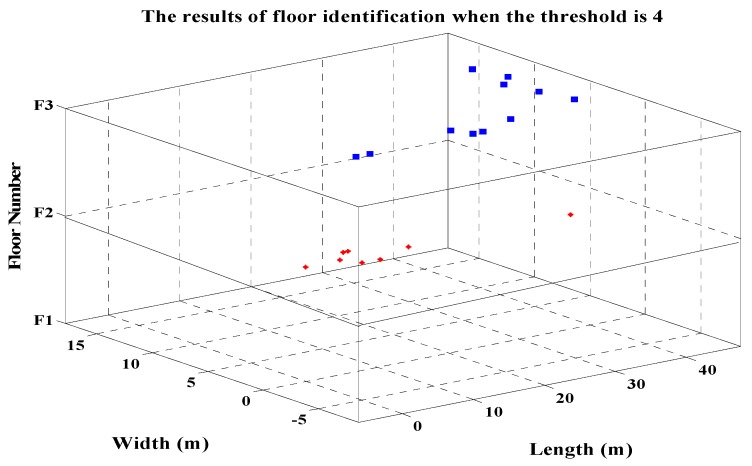
Floor identification results of the moving receiver when the threshold is 4.

When identifying the floor based on MAC repetition rate, the threshold of MAC repetition rate is crucial for the correct identification, with the range of 1 to the dimension of the MAC vector in the database. In order to study the impact of the threshold on floor identification’s accuracy, we let the dimension of the MAC vector be 10 and tested the variation of the floor identification accuracy with the threshold range of [10%, 100%]. We can see from Figure 12 that the accuracy of floor identification can be 100% correct when the threshold is 60%, 70% or 80%. The floor identification rate decreases when the threshold is other values, which makes some test points be identified to wrong floors. As shown in Figure 13, 11 of 19 test points located on the third floor are judged to be on the third floor while the rest to be on the second floor when the threshold is 40%. Therefore, on the stage of floor determination by using the MAC repetition rate, the threshold should be between 60%–80% to ensure the right floor identification completely.

### 5.2. Impact of Clustering Number on Positioning Accuracy

**Figure 14:**
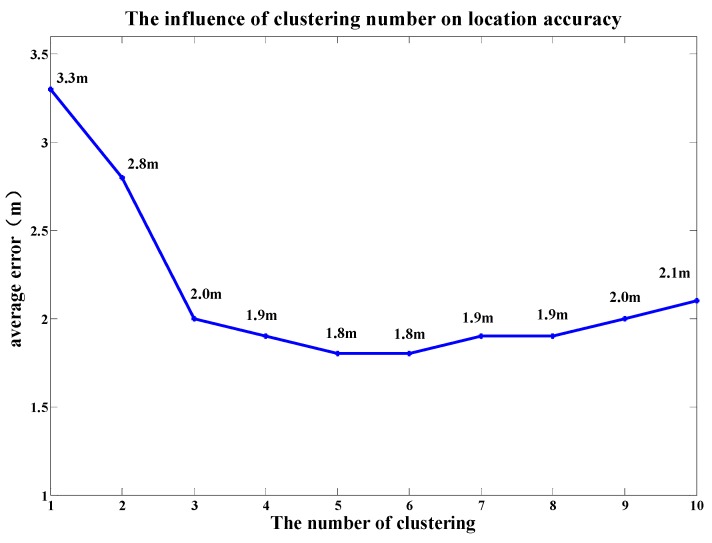
The impact of clustering number on location accuracy.

In order to find whether the clustering number of coarse positioning has an impact on final positioning accuracy, we implemented a test with different clustering numbers with the range of values between 1–10. The test results are shown in Figure 14. When the clustering number is 1, the average position error is 3.3 m. With the increase of the clustering number, the average positioning accuracy improves significantly. When the number of clusters is 5, the positioning error is up to the lowest value of 1.8 m. However, when the number of clusters increases again, the positioning errors begin to increase and the positioning accuracy becomes worse. This is because the reference points near the boundary between two adjacent sub-regions may be clustered into the wrong sub-region.

**Figure 15:**
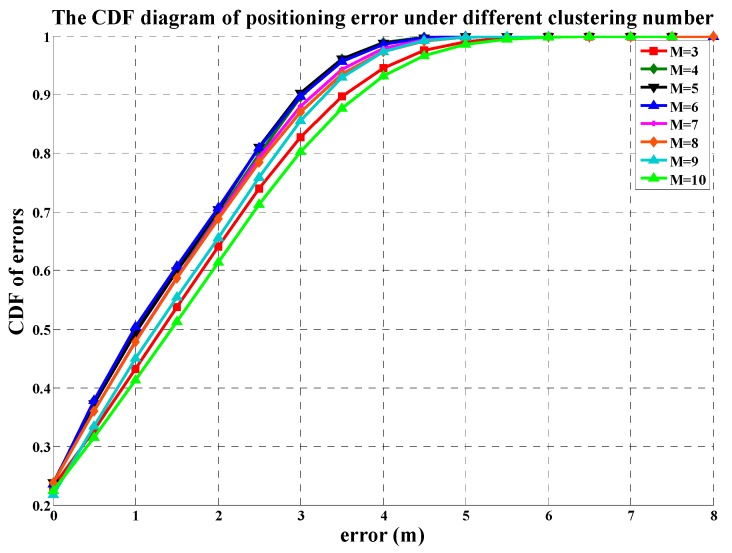
The CDF diagram of positioning error under different clustering numbers.

Figure 15 shows the CDF (Cumulative Distribution Function) diagram of errors when the clustering number is in the range of [3,10]. In the DCF chart, M denotes the clustering number. The more the curve approaches to the upper left, the higher the accuracy of the curve is, on the contrary, the lower right curve has the worst positioning accuracy. When M 4, 5 and 6, the CDF curves of them almost overlap each other. Their probability of location error within 3 m is up to 90% and that within 2 m is up to 70%. When M value is 10, its CDF curve is far away from others, and its probability of location error is only 61% within 2 m. This indicates that the positioning accuracy does not always improve along with the increase of the number of clustering. In addition, theoretically, the increase of the clustering number will increase the complexity of position matching. Therefore, we proposed that the most suitable clustering number is 5 when positioning in real indoor environment.

### 5.3. Impact of Number of APs on Positioning Accuracy

**Figure 16:**
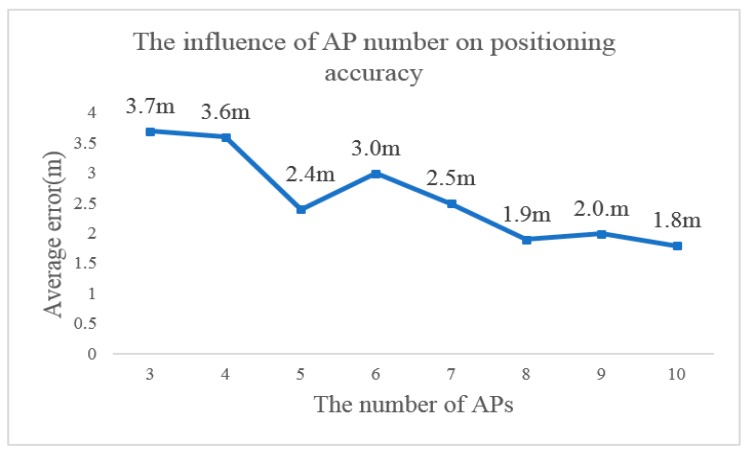
The average positioning accuracy under different reference point densities.

The first factor to consider in determining the number of APs () is the number of APs acceptable to each location point in the location scene, which is affected by the density of AP layout. Only when there are more APs available, can we adopt more APs to locate the receiver. However, according to the principle of the fingerprint localization algorithm, the increase of AP number will increase the storage space requirement of the fingerprint database and reduce the computing efficiency of localization algorithm. In our test area, the receiver at each reference point could access 19 to 129 APs, so we choose the top 10 APs in signal intensity. Based on our established fingerprint database, we tested the number of APs and positioning accuracy, and found that reducing the number of APs will reduce the positioning accuracy as shown in Figure 16. Because our testing fingerprint database only stored 10 of the strongest APs at each reference point, it was given that the change of positioning accuracy when the number of APs varies only from 3 to 10.

### 5.4. Impact of Reference Point Density on Positioning Accuracy

**Figure 17:**
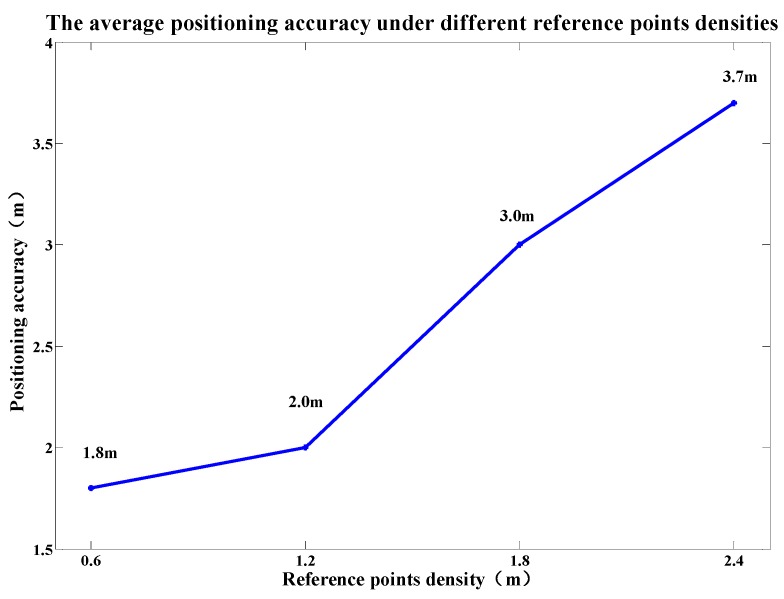
The average positioning accuracy under different reference point densities.

In order to explore the relation between the positioning accuracy and the density of reference points, we laid out a test court in the open area of the first floor of the teaching building J14 with the reference point density of 0.6 m, and then fingerprinting databases of 1.2 m, 1.8 m and 2.4 m were obtained by resampling that of 0.6 m. Figure 17 shows the average positioning accuracy under different reference point densities. When the reference point density is 2.4 m, the positioning error reaches 3.7 m. When the reference point density increases to 1.2 m, the positioning accuracy is greatly improved, with the positioning error of 2.1 m. When the reference point density is 0.6 m, the positioning accuracy is slightly improved, with the positioning error of 1.8 m.

**Figure 18:**
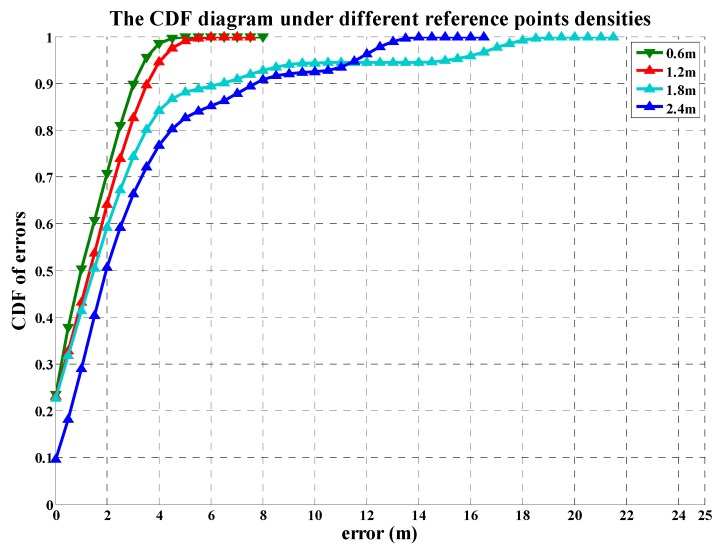
The CDF diagram under different reference point densities.

Figure 18 shows the CDF diagram of the positioning error under different reference points densities. When the reference point density is 2.4 m, the CDF curve is at the bottom and only 50% of the positioning errors are within 2 m. When the reference point density reaches 0.6 m, the CDF curve is located at the top with 71% of the positioning error within 2 m, and the ratio of the positioning error within 3 m is up to 90%. In addition, we can observe that although the reference point density has a positive association with the positioning accuracy, its impact on the positioning accuracy will be unobvious when the reference point density is high to a certain extent. In terms of efficiency, the increase of the reference point density will make the establishment of the fingerprint database complex, so it is the most appropriate choice to set the reference point density as about 1 m.

### 5.5. Change of the MAC Repetition Rate at the Same Point during a Day

**Figure 19:**
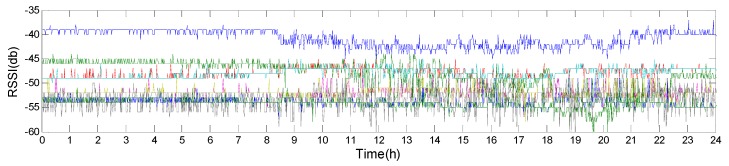
The changes of the RSSIs of the top 10 APs in a day.

In order to test the MAC repetition rate for the same point during different periods of day, we have collected RSSIs and MAC addresses of APs at one-minute intervals, totaling 1440 sets of data. Firstly, let’s see the changes in RSSI over a day shown in Figure 19. From the figure, we can draw some conclusions: (1) The RSSIs of all APs fluctuate greatly from 8:00 am to 23:00 pm while the other time periods are relatively stable for the period from 8:00 am to 23:00 pm is working time and RSSIs are affected greatly by the human activities; (2) The weaker the signal of AP is, the more greatly its RSSI fluctuates, which is related to the path length between the AP and the receiver; (3) The signal intensity of the top 4 APs differ greatly, while the average signal intensity differences of the latter six APs become smaller.

Secondly, let’s see the change of the MAC repetition rate for the same reference point during different periods of the day. Here we give two test results.

**Figure 20:**
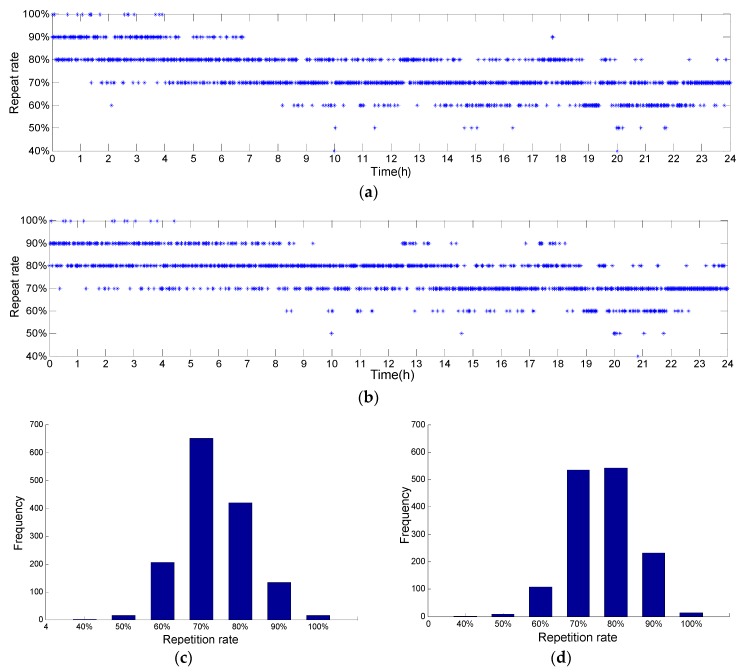
The changes of the MAC repetition rate in a day: (**a**) the datum is at the beginning of 0:00 am; (**b**) the datum is at the beginning of 12:00 am; (**c**) the histogram of the MAC repetition rate of (**a**); (**d**) the histogram of the MAC repetition rate of (**b**).

One is the change of the MAC repetition rate for the same reference point in a day. The mode of the MAC addresses in an hour at the beginning of 0:00 am is taken as datum. The datum vector is composed of MAC addresses of the top 10 APs with the largest occurrence. The change of the MAC repetition rate in a day is as shown in Figure 20a. In addition, to examine the impact of datum selection on results, we also take the mode of the MAC addresses in an hour at the beginning of 12:00 am as datum, and the change of the MAC repetition rate is as shown in Figure 20b. Figure 20c,d shows that the majority of the MAC repetition rates are between 60% and 90%.

When the datum is at the beginning of 0:0 am, the number of the MAC repetition rates of 60%, 70%, 80% and 90% is up to 1408 that accounts for 97.78% of the total 1440. When the datum is at the beginning of 12:00 am, the number of the MAC repetition rates of 60%, 70%, 80% and 90% is up to 1417 that accounts for 98.40% of the total 1440. Detailed statistical results are shown in Table 3 and Table 4.

According to the test results above, we can infer that the change of the MAC repetition rate for the same point is related to that of RSSI during different periods of day, but the detailed relation of them and their impact on indoor positioning accuracy by the change need to be studied further.

**Figure 21:**
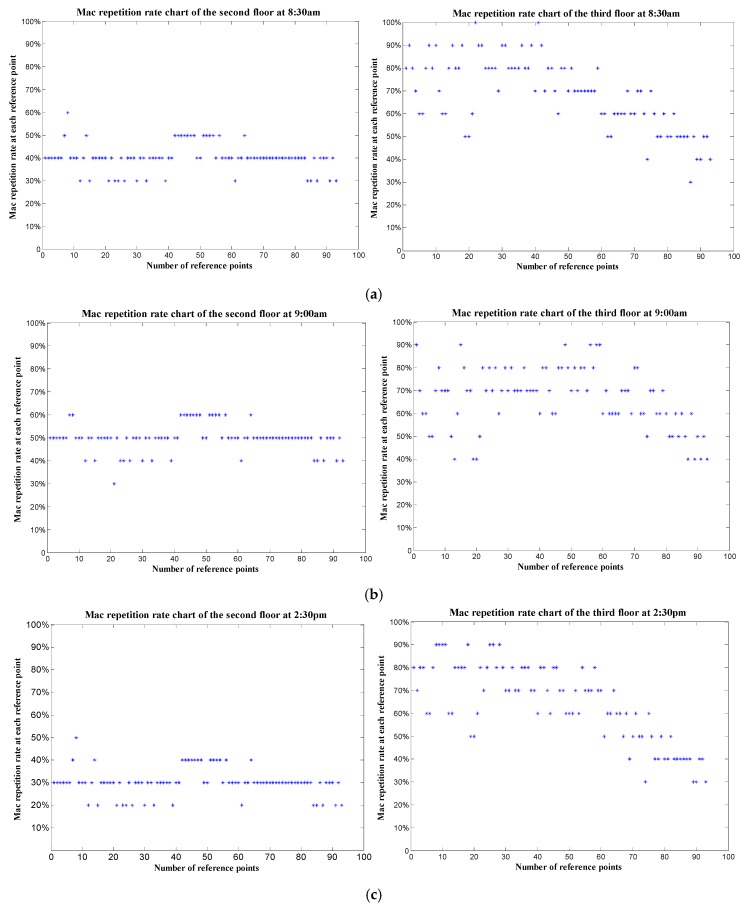

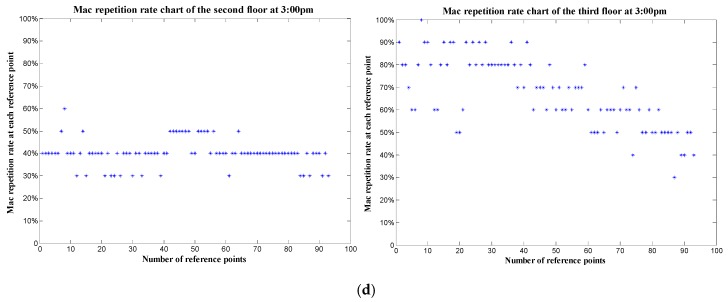
Comparison of MAC repetition rate chart of the second floor and that of the third one: (**a**) at 8:30 am; (**b**) at 9:00 am; (**c**) at 2:30 pm; (**d**) at 3:00 pm.

The other is the change of MAC repetition rate between the test point and reference points on the third and second floor at four different times of a day, which shows the stability of floor identification shown as in Figure 21. The test point is on the third floor.

From Figure 21, it can be seen that at four time points of the day, the Mac repetition rate between the test point and each reference point on the second floor is almost less than 50%, and that between the test point and each reference point on the third floor is almost more than 60%. For the range of the MAC repetition rate changes slightly at these different time points, the results of floor identification are the same at these time points according to Section 5.1.

## 6. Conclusions

As opposed to the existing methods of indoor positioning, the innovation of the proposed method in this paper lies in the introduction of floor identification and the innovative use of MAC addresses of APs, which enables us able to obtain the three-dimensional position of moving receivers in buildings. As MAC addresses of APs are more stable than their RSSI, matching MAC vector is introduced to identify the floor where the moving receiver is located. As long as the appropriate threshold is given, we can obtain higher accuracy in floor identification.

Meanwhile, MAC addresses are also used as the clustering index on the stage of offline clustering reference points to obtain better classification results on the same floor. Clustering reference points based on MAC addresses can not only improve the indoor positioning accuracy for the stability of MAC addresses but also greatly reduce the computational complexity for the reduction of search space of RSSI matching. Our test shows that compared with the case without clustering, the system time consumption is reduced by 29.2% when the number of clusters is 3. In addition, we should choose suitable parameters when implementing the proposed positioning algorithm because the final positioning accuracy is subject to many factors such as clustering number, matching algorithm and reference point density. The positive spatial correlation of spatial distribution of MAC addresses of APs is the key used as the clustering index and significantly benefits indoor three-dimensional positioning.

Although the proposed method in this paper can achieve the indoor three-dimensional location based on fingerprinting database, there are still some aspects deserving further discussion. The biggest challenge is the need to build the fingerprint database beforehand. If the building is very large, it will be a huge task to collect and process MAC and RSSI data of all reference points. Furthermore, it is necessary to modify the fingerprint database in time when APs are changed. Another key problem is the best measurement of the similarity of two vectors. A suitable measurement index can highlight the fingerprint difference of reference points to improve the final location accuracy. In addition, more complex deployments need to be tested such as in multi-floor open atriums where the dispersion of the radio signals across floors or from adjacent buildings would present substantial challenges. Impacts on indoor estimated results by the change of MAC addresses and RSSIs of APs during different periods of day are also a major concern. All those above need to be studied deeply in the future.

## Figures and Tables

**Table 1:** Organization of the fingerprint database.

Reference Points	X	Y	RSSI	MAC	Floor
$A_{t1}$	$X_{t1}$	$Y_{t1}$	$\left\{R_{t11},R_{t12},\ldots ,R_{t1m}\right\}$	$\left\{M_{t11},M_{t12},\ldots ,M_{t1m}\right\}$	$F_{t}$
$A_{t2}$	$X_{t2}$	$Y_{t1}$	$\left\{R_{t21},R_{t22},\ldots ,R_{t2m}\right\}$	$\left\{M_{t21},M_{t22},\ldots ,M_{t2m}\right\}$	$F_{t}$
⋯⋯	⋯⋯	⋯⋯	⋯⋯	⋯⋯	⋯⋯
$A_{ti}$	$X_{ti}$	$Y_{ti}$	$\left\{R_{ti1},R_{ti2},\ldots ,R_{tim}\right\}$	$\left\{M_{ti1},M_{ti2},\ldots ,M_{tim}\right\}$	$F_{t}$

where $A_{ti}$ represents the ID of the reference point i on the floor $F_{t}$; $X_{ti},\;Y_{ti}$ denote the coordinates of reference point $A_{ti}$; $R_{tij}$ and $M_{tij}$ denote respectively the top m of RSSIs and corresponding MAC addresses received by reference point $A_{ti}$; j ranges from 1 to m with the maximum of 10, that is to say, m is 10; $F_{t}$ is the floor number.

**Table 2:** The errors between 18 planned test points and their corresponding estimated points.

**Testing Point**	P1	P2	P3	P4	P5	P6	P7	P8	P9
**Localization Error (m)**	1.3	1.3	0.4	1.2	0.6	3.6	0.6	2.8	1.3
**Testing Point**	P10	P11	P12	P13	P14	P15	P16	P17	P18
**Localization Error (m)**	0.6	3.2	3.1	3.0	2.5	0.6	3.7	0.6	1.5

**Table 3:** The statistical results of MAC repetition rate when the datum is at the beginning of 0:00am.

**MAC Repetition Rate**	40%	50%	60%	70%	80%	90%	100%
**Count**	2	14	206	650	419	133	16

**Table 4:** The statistical results of MAC repetition rate when the datum is at the beginning of 12:00 am.

**MAC Repetition Rate**	40%	50%	60%	70%	80%	90%	100%
**Count**	1	9	107	535	543	232	13
